# Beyond Engagement Alone: Heterogeneous Profiles of Academic Engagement and Mental Well-Being Among Chinese Adolescents

**DOI:** 10.3390/bs16091679

**Published:** 2026-09-17

**Authors:** Yaohua Zhang, Song Chang, Yunyun Huang, Min Xu, Xiaohui Xu, Sufei Xin

**Affiliations:** College of Education, Collaborative Innovation Center for the Mental Health of Youth from the Era of Conversion of New and Old Kinetic Energy Along the Yellow River Basin, Provincial Experimental Teaching Demonstration Center of Psychology, Ludong University, Yantai 264025, China; zhangyh@ldu.edu.cn (Y.Z.); songchang@ldu.edu.cn (S.C.); 3385@ldu.edu.cn (Y.H.); 2881@ldu.edu.cn (M.X.); 3273@ldu.edu.cn (X.X.)

**Keywords:** adolescent, academic engagement, mental well-being, latent profile analysis, school connectedness

## Abstract

Academic engagement and mental well-being are central indicators of adolescent development, yet how these domains jointly characterize heterogeneous patterns of adolescent functioning remains insufficiently understood. This study aimed to identify distinct engagement–well-being configurations among adolescents and to examine factors associated with profile membership and differences in perceived academic achievement across profiles. A sample of 1848 Chinese adolescents in Grades 7, 8, and 10 (ages 12–18 years) completed measures of academic engagement, mental well-being, and demographic, socioeconomic, and psychosocial characteristics. Latent profile analysis identified five distinct configurations, followed by multinomial logistic regression and distal outcome analyses. The resulting profiles were labeled Moderate Distress with Moderate Functioning, Psychologically Stable but Disengaged, Flourishing and Engaged, Distressed but Engaged, and Highly Vulnerable and Disengaged. Notably, relatively high engagement could coexist with psychological vulnerability, suggesting that engagement alone does not indicate optimal functioning. Psychosocial resources were consistently associated with more favorable profiles, while demographic and socioeconomic factors showed more variable associations. Perceived academic achievement differed across profiles, highlighting the value of considering academic engagement and mental well-being jointly when examining adolescents’ perceived academic achievement. These findings underscore the value of a person-centered perspective for understanding adolescent development.

## 1. Introduction

Human flourishing, the state of optimal functioning and well-being across all aspects of an individual’s life, has become a central goal of positive psychology (Keyes, 2007; Lomas et al., 2025; VanderWeele et al., 2026). In Seligman’s authentic happiness theory (Seligman, 2002), engagement was identified as one of three routes to happiness, alongside pleasure and meaning. Engagement was subsequently retained in the PERMA model of well-being (Positive Emotion, Engagement, Relationships, Meaning, and Accomplishment; Seligman, 2011) as one of five independently defined and measurable elements contributing to human flourishing. In school contexts, engagement can be conceptualized as a broad meta-construct encompassing multiple forms of students’ involvement in learning activities and the school community (Wong & Liem, 2022). Depending on the theoretical perspective, engagement may vary in its objects of engagement (e.g., school, classroom, and learning activities) and psychological dimensions (e.g., behavioral, emotional, and cognitive) (Salmela-Aro et al., 2021). This study focuses on academic engagement, which is modeled after the concept of work engagement and defined as a positive affective and motivational state of feeling vigorous, dedicated, and absorbed in learning (Schaufeli et al., 2002). Academic engagement not only facilitates students’ active involvement and sustained investment in learning, thereby promoting academic achievement (Alrashidi et al., 2016; Salanova et al., 2010; Siu et al., 2014; Wong et al., 2024), but also serves as an important developmental strength during adolescence, contributing to positive development and overall well-being (Upadyaya & Salmela-Aro, 2021). Despite substantial evidence linking academic engagement to positive developmental outcomes (Reeve et al., 2025; Suldo & Parker, 2022; Wong et al., 2024), most previous studies have adopted a variable-centered approach, focusing on linear associations between academic engagement and specific developmental indicators. However, such an approach may overlook heterogeneity among adolescents and fail to identify meaningful subgroups with distinct developmental profiles. To address this limitation, the present study adopts a person-centered approach to identify distinct profiles characterized by different patterns of academic engagement and mental well-being.

Although academic engagement is generally linked to better developmental outcomes, high academic engagement does not necessarily correspond to optimal mental well-being, nor does low engagement always co-occur with psychopathological symptoms. In authentic happiness theory, engagement, pleasure, and meaning have been conceptualized and empirically examined as distinguishable orientations to happiness (Peterson et al., 2005). Similarly, the elements of PERMA are not assumed to be interchangeable or reducible to a single component; each contributes to well-being, can be defined and measured independently, and may be pursued for its own sake (Seligman, 2011; Seligman, 2018). Subsequent work has also indicated that different PERMA-related components can be separately targeted and developed through positive psychological interventions (Cabrera & Donaldson, 2024). Thus, although different aspects of positive functioning are related, high functioning in one aspect does not necessarily imply similarly high functioning in the others. Applied to the present study, this separability implies that high academic engagement need not coincide with high life satisfaction, positive affect, or meaning in life. Although academic engagement overlaps conceptually with the engagement element of PERMA through its emphasis on involvement and absorption, it was treated here as a learning-specific motivational construct rather than as a direct measure of PERMA engagement.

A related but distinct theoretical issue concerns the relationship between positive mental well-being and psychological distress. The dual-continua model proposes that positive mental well-being and psychopathological symptoms are related but distinct dimensions rather than opposite ends of a single continuum (Keyes, 2005; Suldo & Doll, 2021; VanderWeele et al., 2026). Accordingly, low psychological distress does not necessarily imply high positive mental well-being, and positive functioning may coexist with elevated distress. Guided by this distinction, positive mental well-being was operationalized in the present study using life satisfaction, positive affect, and meaning in life. These indicators capture complementary evaluative, affective, and eudaimonic aspects of positive functioning, respectively. Psychological distress was operationalized using anxiety symptoms, depressive symptoms, and negative affect, incorporating both symptom-specific and broader affective manifestations of distress. These indicators were not intended to constitute a complete assessment of PERMA or mental well-being. Rather, they were selected to capture theoretically distinguishable positive and negative dimensions of adolescents’ mental well-being while allowing academic engagement to be examined as a separate, learning-specific construct.

Existing person-centered research suggests that these dimensions may be configured differently across individuals. Studies using the PERMA-Profiler (Butler & Kern, 2016) have identified profiles that differ quantitatively in their overall levels of positive well-being, such as low, moderate, and high well-being profiles (F. R. Goodman et al., 2018; Qi et al., 2022). Other studies have identified more differentiated configurations across PERMA dimensions, including profiles in which engagement does not develop in parallel with other aspects of flourishing (Qian et al., 2026). Research informed by the dual-continua model has similarly revealed subgroups characterized by different combinations of positive mental well-being and psychological distress (Suldo & Doll, 2021). In the academic domain, Salmela-Aro et al. (2016) identified distinct student engagement profiles, including highly engaged and burned-out groups, and importantly, an “engaged-exhausted” profile characterized by the coexistence of high engagement and burnout symptoms. These findings suggest that academic engagement may not always correspond to optimal well-being, as positive academic functioning can coexist with domain-specific negative experiences. However, previous person-centered studies have typically examined these dimensions within separate frameworks, focusing either on positive mental well-being (e.g., PERMA framework) or overall mental well-being (e.g., the dual-continua model). Whether academic engagement can be jointly characterized alongside positive mental well-being and psychopathological symptoms remains unclear.

The Job Demands–Resources (JD–R) model provides a complementary framework for interpreting such heterogeneity. Whereas authentic happiness theory and the PERMA model support the separability of engagement and other positive functioning indicators, and the dual-continua model distinguishes positive mental well-being from psychological distress, the JD–R model offers a contextual lens for understanding how different engagement–well-being configurations may be associated with personal and environmental resources and demands. According to this model, engagement represents a motivational state that is fostered by adequate resources, whereas excessive demands may undermine well-being and contribute to psychological distress (Bakker et al., 2014, 2023). When extended to school contexts, this perspective suggests that academic engagement may not uniformly correspond to mental well-being, but may vary depending on the broader balance between available resources and psychological demands (Salmela-Aro et al., 2022).

Despite these theoretical and empirical advances, an important gap remains. Previous person-centered studies have generally examined academic engagement, positive mental well-being, or psychopathological symptoms in separate profiling frameworks. Consequently, insufficient attention has been paid to how these three domains are jointly configured within adolescents, including whether similar levels of academic engagement may coexist with markedly different combinations of positive psychological functioning and psychological distress. Examining these domains jointly may therefore reveal meaningful within-person heterogeneity that would be obscured when academic engagement or mental health is considered in isolation. This question may be particularly relevant in the Chinese educational context. Chinese adolescents experience highly competitive schooling and high-stakes educational transitions, including the Zhongkao and Gaokao, while academic achievement is often strongly emphasized by both families and schools (Sun et al., 2013).

The present study therefore adopted a person-centered approach to examine the joint configurations of academic engagement, positive mental well-being, and psychological distress among Chinese adolescents. Specifically, we first used latent profile analysis to identify subgroups characterized by distinct patterns across these domains. We then examined demographic, socioeconomic, and psychosocial characteristics as covariates of profile membership and compared perceived academic achievement across the identified profiles. Guided by the dual-continua and JD–R perspectives, together with previous findings concerning engaged-exhausted students (Salmela-Aro et al., 2016), we expected both broadly concordant and discordant configurations to emerge. In particular, we anticipated a configuration combining relatively high academic engagement with elevated psychological distress, as well as a configuration combining relatively low academic engagement with comparatively preserved psychological functioning. We also considered it plausible that more broadly adaptive and vulnerable configurations would emerge. However, because latent profile analysis is exploratory with respect to profile enumeration, we did not specify the exact number, prevalence, or detailed form of the profiles a priori.

## 2. Materials and Methods

### 2.1. Participants

Participants were recruited from three public schools in Anqiu City, Shandong Province, China. Schools were selected through convenience sampling, and all students in the selected classes within these schools were invited to participate. Data collection was conducted during regular school hours, with trained research assistants administering paper-and-pencil questionnaires in classroom settings. Prior to data collection, informed consent was obtained from school administrators, parents/guardians, and the participating adolescents. Participants were explicitly informed that their participation was voluntary, that they could withdraw at any time without penalty, and that their responses would remain strictly confidential. The study protocol was approved by the Institutional Review Board (IRB) of Ludong University (Approval No. LDU-CE0024112001).

A total of 1848 adolescents participated in the study, comprising students from two junior high schools (*n* = 625, 33.82%; *n* = 559, 30.25%) and one senior high school (*n* = 664, 35.93%). Among participants who reported valid sex information (*n* = 1826), 1001 were male (54.82%) and 825 were female (45.18%). Participants ranged in age from 12 to 18 years, with a mean age of 14.39 years (*SD* = 1.41). Regarding grade distribution, the sample consisted of students from Grade 7 (*n* = 801, 43.34%), Grade 8 (*n* = 383, 20.73%), and Grade 10 (*n* = 664, 35.93%). Grade 9 students were excluded from participation because they were engaged in intensive preparation for the senior high school entrance examination (locally known as Zhongkao) during the data collection period.

### 2.2. Measures

The positive dimension of mental well-being was represented by life satisfaction, positive affect, and meaning in life, whereas psychological distress was represented by anxiety symptoms, depressive symptoms, and negative affect.

**Anxiety.** Anxiety was assessed using the 20-item Self-Rating Anxiety Scale (Zung, 1971). The scale assesses cognitive, motor, autonomic, and central nervous system manifestations of anxiety, with all items combined to produce an overall anxiety score. A sample item is “I feel more nervous and anxious than usual.” Participants reported the frequency of these experiences during the past week on a 4-point scale ranging from 1 (rarely or none of the time) to 4 (most or all of the time), with higher scores indicating greater anxiety symptoms. The Chinese version has demonstrated acceptable psychometric properties among Chinese students (Pang et al., 2019). The scale demonstrated acceptable internal consistency in the present study (Cronbach’s α = 0.83).

**Depression.** Depressive symptoms were assessed using the 20-item Center for Epidemiologic Studies Depression Scale (Radloff, 1977). The scale includes items covering depressed affect, positive affect, somatic and retarded activity, and interpersonal difficulties, with all 20 items combined to produce an overall depression score. A sample item is “I felt depressed.” Participants reported the frequency of these experiences during the past week on a 4-point scale ranging from 1 (rarely or none of the time) to 4 (most or all of the time), with higher scores indicating greater depressive symptoms. The scale has been validated among Chinese adolescents (Zhu et al., 2021). It demonstrated excellent internal consistency in the present study (Cronbach’s α = 0.90).

**Life Satisfaction.** Life satisfaction was assessed using the unidimensional five-item Satisfaction With Life Scale (Diener et al., 1985). A sample item is “I am satisfied with my life.” Participants rated each item on a 7-point scale ranging from 1 (strongly disagree) to 7 (strongly agree), with higher scores indicating greater life satisfaction. The scale has been validated among Chinese adolescents (W. Chen et al., 2017). It demonstrated acceptable internal consistency in the present study (Cronbach’s α = 0.86).

**Meaning in Life.** Meaning in life was assessed using the five-item presence of meaning subscale of the Meaning in Life Questionnaire (Steger et al., 2006). Although the original questionnaire comprises two dimensions—presence of meaning and search for meaning—only the presence of meaning dimension was included in the present study. A sample item is “I understand my life’s meaning.” Participants rated each item on a 7-point scale ranging from 1 (not at all true of me) to 7 (completely true of me), with higher scores indicating a stronger sense that their lives were meaningful. The MLQ has been validated among Chinese adolescents (X. Wang, 2013). The presence of meaning subscale demonstrated acceptable internal consistency in the present study (Cronbach’s α = 0.86).

**Positive and Negative Affect.** Positive and negative affect were assessed using the 20-item Positive and Negative Affect Schedule (PANAS; Watson et al., 1988). This two-dimensional measure comprises 10 items assessing positive affect (e.g., “enthusiastic”) and 10 items assessing negative affect (e.g., “distressed”). Participants indicated the extent to which they had experienced each affective state during the past month on a 5-point scale ranging from 1 (almost none) to 5 (very much), with higher scores indicating higher levels of the corresponding type of affect. The scale has been validated in Chinese samples (Huang et al., 2003). The positive- and negative-affect subscales demonstrated acceptable internal consistency in the present study (Cronbach’s α = 0.83 and 0.89, respectively).

**Academic Engagement.** Academic engagement was assessed using the nine-item short form of the Utrecht Work Engagement Scale for Students (Schaufeli et al., 2002, 2006). This three-dimensional scale comprises three items assessing vigor (e.g., “When I study, I feel like I am bursting with energy”), three items assessing dedication (e.g., “I am enthusiastic about my studies”), and three items assessing absorption (e.g., “I am immersed in my studies”). Participants rated each item on a 7-point scale ranging from 1 (never) to 7 (always), with higher scores indicating higher levels of academic engagement. The scale has been validated among Chinese students (Wei et al., 2025). The overall scale demonstrated excellent internal consistency in the present study (Cronbach’s α = 0.94).

**Parental Education.** Parental education was assessed by asking adolescents to report whether their father and mother had received higher education. Education level was dichotomized into 0 (no higher education; high school or below) and 1 (higher education; associate degree or above). Higher education included associate degree, undergraduate, and postgraduate education.

**Economic Strain.** Economic strain was assessed using a measure adapted from Wadsworth and Compas (2002). This unidimensional scale consists of four items assessing financial difficulties related to clothing, food, housing, and entertainment (e.g., “My family does not have enough money to buy new clothes”). Participants rated each item on a 5-point scale ranging from 1 (never) to 5 (always), with higher scores indicating greater perceived economic strain. The scale has been validated among Chinese adolescents (J. Wang et al., 2010). It demonstrated good internal consistency in the present study (Cronbach’s α = 0.80).

**Subjective Social Status.** Subjective social status was assessed using the MacArthur Scale of Subjective Social Status–Youth Version (E. Goodman et al., 2001). It includes two indicators reflecting adolescents’ perceived social standing in different contexts. The first indicator assessed subjective family social status (family SSS), with adolescents rating their family’s position in society on a 10-point ladder ranging from 1 (lowest social status) to 10 (highest social status). The second indicator assessed subjective personal social status (personal SSS), with adolescents rating their own social standing relative to their schoolmates on a 10-point ladder ranging from 1 (lowest social status) to 10 (highest social status). The scale has been validated among Chinese adolescents (M. Hu et al., 2012). Higher scores indicated higher perceived social status.

**Implicit Theories of Intelligence.** Implicit theories of intelligence were assessed using the scale developed by Dweck (1999). This two-dimensional scale consists of four items assessing entity beliefs (e.g., “You have a certain amount of intelligence, and you cannot really do much to change it”) and four items assessing incremental beliefs (e.g., “You can always substantially change how intelligent you are”). Participants rated each item on a 6-point scale ranging from 1 (strongly disagree) to 6 (strongly agree), with higher scores indicating stronger endorsement of the corresponding belief. The scale has been validated among Chinese adolescents (Q. Wang & Ng, 2012). The entity and incremental belief subscales demonstrated good internal consistency in the present study (Cronbach’s α = 0.94 and 0.84, respectively).

**School Connectedness.** School connectedness was assessed using the measure developed by McNeely et al. (2002). This unidimensional scale consists of five items (e.g., “I feel close to people at this school”). Participants rated each item on a 5-point scale ranging from 1 (strongly disagree) to 5 (strongly agree), with higher scores indicating stronger feelings of connection and belonging to school. The scale has been validated among Chinese adolescents (He et al., 2019). It demonstrated good internal consistency in the present study (Cronbach’s α = 0.87).

**Perceived Academic Achievement.** Perceived academic achievement was assessed using three self-report items that asked students to evaluate their academic performance in Chinese, Mathematics, and English. Specifically, students responded to the statements, “I think my achievement in Chinese is…,” “I think my achievement in mathematics is…,” and “I think my achievement in English is….” Each item was rated on a five-point Likert scale ranging from 1 (very poor) to 5 (very good), with higher scores indicating higher levels of perceived academic achievement. The mean score across the three items was calculated, with higher scores reflecting more favorable self-perceived academic achievement. This approach of assessing academic achievement through students’ subjective evaluations of their own academic performance has been adopted in previous studies (Y. Hu et al., 2025; Sticca et al., 2017), demonstrating its utility for capturing students’ perceived academic competence and achievement-related performance. The three-item measure showed modest internal consistency in the present study (Cronbach’s α = 0.60).

### 2.3. Statistical Analysis

All the statistical analyses were conducted using R (Version 4.5.3; R Core Team, 2026) and Mplus (Version 9; Muthén & Muthén, 1998–2025). Data management, descriptive statistics, correlation analyses, missing data diagnostics, and reliability analyses were performed in R using the tidyverse (Version 2.0.0; Wickham et al., 2019), psych (Version 2.6.5; Revelle, 2026), and naniar (Version 1.1.0; Tierney & Cook, 2023) packages.

A joint latent profile analysis (LPA) was conducted to identify subgroups of adolescents characterized by distinct combinations of academic engagement, positive mental well-being, and psychopathological symptoms. Before model estimation, all the profile indicators were standardized as *z* scores (*M* = 0, *SD* = 1) to place them on a common metric. The LPA proceeded in two stages, with profile enumeration followed by classification diagnostics. In the present study, candidate models were estimated in batches through the tidyLPA package (Version 2.0.2; Rosenberg et al., 2018) in R, which interfaces with Mplus for model estimation. All the candidate models were estimated using robust maximum likelihood (MLR), with TYPE = MIXTURE specified in Mplus. Methodological guidance on class enumeration in mixture models with nested data remains limited (Musci et al., 2024). During profile enumeration, the TYPE = MIXTURE COMPLEX option was not used to adjust standard errors for classroom clustering.

In the first stage, alternative variance–covariance structures were examined to determine both the number of profiles and the appropriate model specification (Pastor et al., 2007). Models containing one to six profiles were estimated under each of the six parameterizations available in tidyLPA, yielding 36 candidate models. These parameterizations included Model 1 with equal variances and zero covariances, Model 2 with varying variances and zero covariances, Model 3 with equal variances and equal covariances, Model 4 with varying variances and equal covariances, Model 5 with equal variances and varying covariances, and Model 6 with varying variances and varying covariances. Here, equal and varying indicated whether corresponding within-profile variance or covariance parameters were constrained to equality across profiles or freely estimated across profiles, respectively. Profile enumeration was guided by the Akaike information criterion (AIC), consistent Akaike information criterion (CAIC), Bayesian information criterion (BIC), sample-size-adjusted BIC (aBIC), approximate weight of evidence criterion (AWE), Vuong–Lo–Mendell–Rubin likelihood ratio test (VLMR), adjusted Lo–Mendell–Rubin likelihood ratio test (aLMR), and bootstrapped likelihood ratio test (BLRT), together with profile sizes, parsimony, and theoretical interpretability. Lower information-criterion values indicated greater support for a candidate model. The VLMR, aLMR, and BLRT compared a *k*-profile solution with the corresponding (*k* − 1)-profile solution within the same parameterization, with significant results favoring the *k*-profile solution. Given the nested structure of the data, particular attention was paid to BIC and CAIC, informed by simulation evidence suggesting their relatively favorable class enumeration performance in growth mixture models that ignore higher-level nesting, particularly with larger samples and/or lower intraclass correlations (Q. Chen et al., 2017). Nevertheless, no single criterion was treated as decisive. The final solution was selected through an integrated evaluation of statistical and substantive evidence (Masyn, 2013; Sorgente et al., 2025/2025; Spurk et al., 2020).

In the second stage, the classification quality of the selected solution was evaluated using entropy, modal class assignment proportions (mcaP_k_), class-specific average posterior probabilities (AvePP_k_), and odds of correct classification (OCC_k_). Entropy values closer to 1 indicated greater overall classification precision, with values of 0.70 and 0.80 used as general guides for acceptable and good classification quality, respectively (Moore et al., 2025). AvePP_k_ values above 0.70 and OCC_k_ values greater than 5.0 were considered indicative of adequate classification accuracy and profile separation (Masyn, 2013). Agreement between the model-estimated profile proportions and mcaP_k_ values was also examined to assess potential classification bias.

Following these two stages, auxiliary analyses were conducted directly in Mplus to examine associations between candidate covariates and profile membership and to compare perceived academic achievement across profiles. Classification uncertainty was addressed using the built-in R3STEP and BCH procedures implemented through the AUXILIARY option in Mplus, whereas the dependence of students within classrooms was addressed through cluster-robust estimation. A unique classroom identifier was constructed by combining school, grade, and class, yielding 36 classroom clusters. Both auxiliary analyses were estimated using MLR with TYPE = MIXTURE COMPLEX and the classroom identifier specified as the clustering variable. This specification adjusted standard errors, confidence intervals, and significance tests for classroom clustering.

Associations between candidate covariates and profile membership were examined using multinomial logistic regression with the R3STEP procedure in Mplus, which accounts for uncertainty in profile classification (Asparouhov & Muthén, 2014). Reference profiles were varied as needed to obtain the relevant comparisons. Logit coefficients, odds ratios, and significance levels were reported, with 95% CIs provided for focal comparisons in the text. The pairwise covariate contrasts conducted via the R3STEP procedure were treated as exploratory and were not adjusted for multiplicity. Perceived academic achievement was examined as a continuous distal outcome using the BCH procedure in Mplus, which accounts for classification uncertainty without allowing the distal outcome to alter the definition of the latent profiles (Bolck et al., 2004). An overall test of mean differences was followed by pairwise comparisons to identify specific differences between profiles. Both the overall and pairwise tests incorporated the classroom-level cluster adjustment described above. To control the false discovery rate at 0.05, the Benjamini–Hochberg procedure was applied across all pairwise comparisons for the distal outcome.

Missing data were handled differently depending on the role of the variables in the analyses. Little’s MCAR test was conducted to examine the missing data mechanism for the profile indicators. The result indicated that missingness was consistent with the assumption of missing completely at random (MCAR), χ^2^(34) = 28.90, *p* = 0.72. Missing values on the profile indicators were handled using full information maximum likelihood (FIML) estimation under the missing-at-random (MAR) assumption. This approach allows cases with partially missing profile indicators to be retained in the latent profile analysis by using all available information. For covariates and the distal outcome, cases with missing values were excluded using listwise deletion. The missing rate for these variables was low (maximum = 3.24%), and therefore listwise deletion was considered unlikely to substantially affect the results (Tabachnick & Fidell, 2019).

## 3. Results

### 3.1. Descriptive Statistics

Descriptive statistics and correlations for all the study variables are presented in Table 1. The seven indicators used to establish latent profiles of mental well-being and academic engagement showed considerable variability. Participants reported relatively low levels of anxiety (*M* = 1.64, *SD* = 0.40), depression (*M* = 1.64, *SD* = 0.50), and negative affect (*M* = 2.12, *SD* = 0.77), whereas they reported relatively higher levels of life satisfaction (*M* = 4.54, *SD* = 1.44), positive affect (*M* = 3.21, *SD* = 0.87), meaning in life (*M* = 4.18, *SD* = 1.53), and academic engagement (*M* = 4.51, *SD* = 1.50).

The variables examined as covariates of latent profile membership included demographic characteristics, family socioeconomic indicators, implicit beliefs, and school connectedness. Among these variables, participants reported moderate levels of family SSS (*M* = 5.40, *SD* = 1.44), personal SSS (*M* = 5.65, *SD* = 1.63), incremental beliefs (*M* = 3.41, *SD* = 1.22), and school connectedness (*M* = 3.88, *SD* = 0.88), whereas they reported relatively low levels of entity beliefs (*M* = 2.80, *SD* = 1.40). Perceived academic achievement, which was examined as a distal outcome of latent profile membership, had a mean score of 2.82 (*SD* = 0.79).

Correlation analyses indicated that the indicators of mental well-being and academic engagement were significantly correlated in theoretically expected directions. Specifically, negative indicators of mental well-being (i.e., anxiety, depression, and negative affect) were positively correlated with each other and negatively correlated with positive indicators of mental well-being (i.e., life satisfaction, positive affect, and meaning in life). In contrast, positive indicators of mental well-being were positively associated with academic engagement, whereas negative indicators of mental well-being were negatively associated with academic engagement.

### 3.2. Joint Latent Profiles of Mental Well-Being and Academic Engagement

A total of 36 candidate LPA models, comprising six variance–covariance specifications with one to six profiles, were evaluated (Table 2). The information criteria provided stronger support for specifications allowing within-profile covariances than for those fixing covariances at zero. Specifically, Models 1 and 2 generally yielded higher BIC and CAIC values than Models 3–6 at corresponding profile counts. Among the specifications allowing covariances, however, the criteria did not converge on a single solution. The lowest BIC and CAIC were obtained for the six-profile solution under Model 4, whereas the lowest AWE was obtained for its three-profile solution. Under Models 5 and 6, CAIC increased beyond three profiles, and AWE increased beyond two profiles, indicating that additional complexity was not consistently supported by these penalized fit criteria.

The likelihood ratio tests also provided mixed evidence regarding profile enumeration. The BLRT was significant for all comparisons between adjacent profile solutions across the six specifications (all *p*s < 0.001), providing no clear stopping point within the range examined. The VLMR and aLMR supported five profiles over four, but not six over five, under Models 1 and 2. Under Models 4, 5, and 6, neither test supported increases beyond three, four, and two profiles, respectively. Under Model 3, both tests remained significant through six profiles. These results concerned increases in profile number within each specification rather than direct comparisons between variance–covariance structures.

Classification precision and model parsimony were therefore considered alongside these findings. Across the two- to six-profile solutions, entropy ranged from 0.811 to 0.843 under Model 3, compared with 0.520 to 0.778 under Models 4–6. Model 3 also required fewer parameters than Models 4–6 at corresponding profile counts because both indicator variances and covariances were constrained to equality across profiles. Taken together, the improvement in information criteria relative to Models 1 and 2, the more parsimonious structure relative to Models 4–6, and the consistently clearer classification supported retaining Model 3 for closer examination of profile number. This represented a balance between fit, parsimony, and classification utility, rather than a claim that Model 3 minimized the information criteria across all candidates (Nylund-Gibson & Choi, 2018).

Within Model 3, AIC, CAIC, BIC, and aBIC decreased as profiles were added, whereas AWE reached its minimum at four profiles (31,298.801). Increasing the number of profiles from four to five reduced AIC to 30,257.931, CAIC to 30,694.895, BIC to 30,627.896, and aBIC to 30,415.038. All three likelihood ratio tests supported this increase (VLMR *p* < 0.001, aLMR *p* = 0.001, and BLRT *p* < 0.001). Adding a sixth profile further reduced these information criteria, and the likelihood ratio tests remained significant (VLMR *p* = 0.032, aLMR *p* = 0.034, and BLRT *p* < 0.001). However, AWE increased from 31,332.859 for five profiles to 31,398.199 for six profiles, indicating disagreement among the statistical criteria.

Examination of the profile configurations provided additional grounds for choosing between these solutions. The six-profile solution identified an additional profile characterized by markedly elevated negative affect alongside otherwise relatively favorable psychological functioning and academic engagement. This profile contained only 25 students (1.35% of the sample) based on most likely class membership and was distinguished primarily by a single indicator. It is generally recommended that profile sizes be at least 5–8% of the sample (Moore & Quartiroli, 2026). Its small size raised concerns about the precision of profile-specific estimates and subsequent subgroup comparisons, while its pattern of differentiation warranted caution regarding the substantive value of retaining an additional profile. These considerations were evaluated alongside the statistical improvements, consistent with recommendations to consider class size, parsimony, and interpretability when assessing additional profiles (Sorgente et al., 2025/2025). On balance, the five-profile solution under Model 3 was selected for subsequent analyses. The solution had an entropy value of 0.811, and its smallest profile contained 98 students (5.30% of the sample) based on most likely class membership. The best log-likelihood value (*LL* = −15,061.965) was obtained from 20 distinct sets of random starting values, supporting the numerical reproducibility of the selected solution.

Classification diagnostics were examined to assess how confidently students could be assigned to the five profiles (Table 3). Model-estimated proportions summarize the relative size of each profile while allowing for uncertainty in membership. In contrast, modal class assignment proportions (mcaP_k_) count each student in the profile with their highest posterior probability. The two sets of proportions differed by less than one percentage point for every profile. All modal proportions also fell within the corresponding asymmetric 95% bootstrap confidence intervals. These findings indicated close agreement between the model-estimated profile sizes and those obtained by assigning each student to their most likely profile.

Average posterior probabilities (AvePP_k_) provided a complementary assessment of assignment certainty within each profile. This index summarizes the average probability of membership in a profile among students assigned to that profile. Values closer to 1 indicate stronger model-based support for these assignments. In the present study, AvePP_k_ values ranged from 0.79 to 0.92, exceeding the recommended benchmark of 0.70 for all the profiles. Thus, students assigned to each profile generally had relatively high estimated probabilities of belonging to it.

The odds of correct classification (OCC_k_) assessed classification relative to a baseline that uses profile proportions alone. Specifically, this index compares model-based classification odds with those associated with random assignment according to the estimated profile proportions. OCC_k_ values ranged from 9.30 to 106.43, exceeding the recommended benchmark of 5.0 across all the profiles. Taken together, these diagnostics supported adequate classification precision. Nevertheless, profile membership remained uncertain, and this uncertainty was accounted for in the subsequent auxiliary analyses.

**Figure 1:**
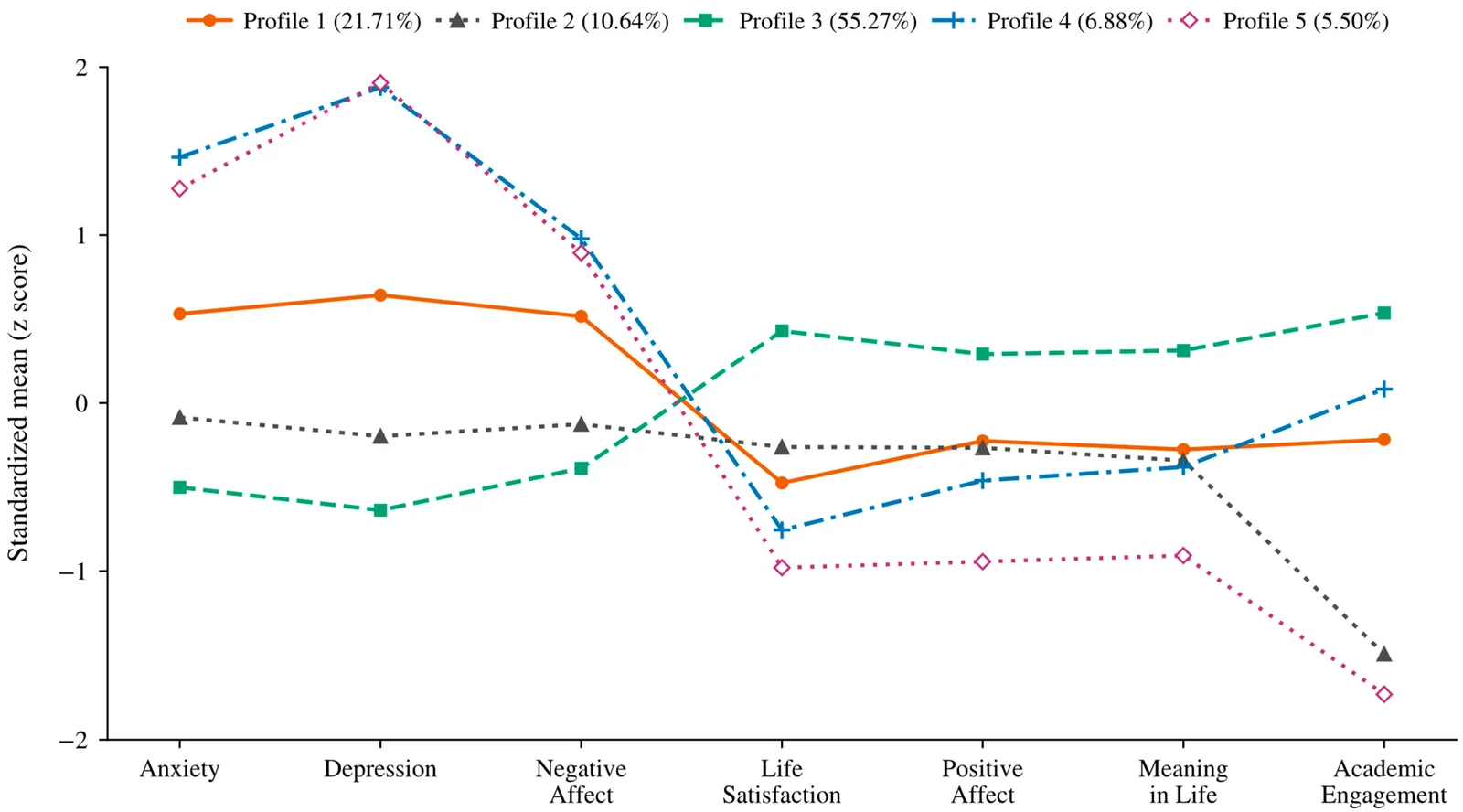
Joint profiles of academic engagement and mental well-being.

To characterize the heterogeneity underlying the five latent profiles, pairwise comparisons of profile-specific means across the seven indicators were conducted using the MODEL CONSTRAINT command in Mplus. Statistical significance was evaluated based on 95% percentile bootstrap confidence intervals derived from 1000 bootstrap resamples, with intervals excluding zero indicating significant differences between profiles. Profile labels were assigned based on the overall configuration of academic engagement and mental well-being (Figure 1). To further inform profile interpretation, Table A1 in Appendix A presents pairwise differences in model-estimated means across the five profiles for each indicator, together with 95% bootstrap confidence intervals based on 1000 resamples.

Profile 3, accounting for the largest proportion of the sample (55.27%), was labeled the Flourishing and Engaged Profile. This profile showed a consistently favorable configuration across all seven indicators. Below-average anxiety, depressive symptoms, and negative affect (*M*s ranging from −0.64 to −0.39) coexisted with above-average life satisfaction, positive affect, and meaning in life (*M*s ranging from 0.29 to 0.43). Academic engagement was also above average (*M* = 0.54). Its defining feature was the combination of relatively low psychological distress, high positive mental well-being, and strong academic engagement.

Profile 1 (21.71%) was labeled the Moderate Distress with Moderate Functioning Profile. This profile showed moderately elevated anxiety, depressive symptoms, and negative affect, with standardized means ranging from 0.52 to 0.64. Positive mental well-being was somewhat below average, particularly life satisfaction (*M* = −0.48), whereas academic engagement was only slightly below average (*M* = −0.22). Elevated psychological distress was therefore accompanied by relatively modest reductions in positive functioning, without the marked disengagement observed in Profiles 2 and 5.

Profile 2 (10.64%) was labeled the Psychologically Stable but Disengaged Profile. Its most distinctive characteristic was low academic engagement (*M* = −1.49). In contrast, anxiety, depressive symptoms, and negative affect were close to or slightly below the sample average (*M*s ranging from −0.20 to −0.09). Life satisfaction, positive affect, and meaning in life were also slightly below average. Thus, pronounced academic disengagement was not accompanied by the elevated psychological distress observed in Profiles 4 and 5. Nevertheless, this group lacked the above-average positive mental well-being that characterized the Flourishing and Engaged Profile.

Profile 4 (6.88%) was labeled the Distressed but Engaged Profile. This profile combined elevated anxiety (*M* = 1.46), depressive symptoms (*M* = 1.88), and negative affect (*M* = 0.98) with academic engagement close to the sample average (*M* = 0.08). Although anxiety and depressive symptoms did not differ significantly from those in Profile 5, academic engagement was significantly higher. Positive mental well-being remained below average, but positive affect and meaning in life were also significantly higher than in Profile 5. This configuration highlighted the coexistence of substantial psychological distress with comparatively higher academic engagement.

Profile 5 (5.50%) was labeled the Highly Vulnerable and Disengaged Profile. This profile showed difficulties across both psychological and academic domains. Elevated anxiety (*M* = 1.28) and depressive symptoms (*M* = 1.91) were accompanied by the lowest estimated means for life satisfaction, positive affect, and meaning in life (*M*s ranging from −0.98 to −0.91). Academic engagement was also markedly below average (*M* = −1.73), although it did not differ significantly from that in Profile 2. Unlike Profile 2, however, this group combined low engagement with heightened psychological distress and low positive mental well-being, reflecting a broadly unfavorable configuration across the assessed domains.

### 3.3. Covariates of Latent Profile Membership

Associations of demographic, socioeconomic, and psychosocial characteristics with latent profile membership were examined using multinomial logistic regression with the automated three-step (R3STEP) procedure in Mplus. All 10 covariates were entered simultaneously, and analyses used robust maximum likelihood estimation with TYPE = MIXTURE COMPLEX to account for clustering within 36 classrooms. The auxiliary analyses included 1729 adolescents after excluding 119 participants with missing covariate data. Primary comparisons focused on Profile 3 (Flourishing and Engaged Profile), followed by additional comparisons between theoretically relevant profiles. Associations were interpreted using odds ratios (ORs) and their corresponding 95% confidence intervals (CIs). The full results are presented in Table A2.

Regarding demographic characteristics, female adolescents had lower odds than male adolescents of belonging to Profile 2 (Psychologically Stable but Disengaged Profile) rather than Profile 3, *OR* = 0.331, 95% CI [0.199, 0.550], *p* < 0.001. Sex did not significantly differentiate the remaining profiles from Profile 3. Age showed selective associations with profile membership. Older adolescents had higher odds of belonging to Profile 4 (Distressed but Engaged Profile), *OR* = 1.255, 95% CI [1.020, 1.545], *p* = 0.032, and Profile 5 (Highly Vulnerable and Disengaged Profile), *OR* = 1.329, 95% CI [1.019, 1.734], *p* = 0.036, rather than Profile 3.

Regarding socioeconomic characteristics, higher paternal education was associated with higher odds of belonging to Profile 4 (Distressed but Engaged Profile) rather than Profile 3, *OR* = 2.102, 95% CI [1.040, 4.249], *p* = 0.038. Paternal education did not significantly distinguish the other profiles from Profile 3, and maternal education did not significantly differentiate any profile from Profile 3. Greater economic strain was associated with higher odds of belonging to several profiles relative to the Flourishing and Engaged Profile. Specifically, economic strain was positively associated with membership in Profile 1 (Moderate Distress with Moderate Functioning Profile), *OR* = 1.880, 95% CI [1.166, 3.031], *p* = 0.010; Profile 4 (Distressed but Engaged Profile), *OR* = 2.467, 95% CI [1.513, 4.022], *p* < 0.001; and Profile 5 (Highly Vulnerable and Disengaged Profile), *OR* = 1.855, 95% CI [1.142, 3.015], *p* = 0.013, rather than Profile 3. Economic strain did not significantly distinguish Profile 2 (Psychologically Stable but Disengaged Profile) from Profile 3.

Subjective social status indicators showed different patterns of association. Higher personal SSS was associated with higher odds of belonging to Profile 3 rather than Profile 1 (Moderate Distress with Moderate Functioning Profile), *OR* = 1.164, 95% CI [1.015, 1.336], *p* = 0.030. It was also associated with lower odds of belonging to Profile 4 (Distressed but Engaged Profile), *OR* = 0.767, 95% CI [0.617, 0.954], *p* = 0.017, and Profile 5 (Highly Vulnerable and Disengaged Profile), *OR* = 0.784, 95% CI [0.615, 0.999], *p* = 0.049, rather than Profile 3. Personal SSS did not significantly distinguish Profile 2 from Profile 3. Family SSS did not significantly differentiate any of the other profiles from Profile 3.

Regarding psychosocial characteristics, entity and incremental beliefs showed contrasting associations with profile membership. Stronger entity beliefs were associated with lower odds of belonging to Profile 3 rather than Profile 1 (Moderate Distress with Moderate Functioning Profile), *OR* = 0.828, 95% CI [0.713, 0.963], *p* = 0.014; Profile 2 (Psychologically Stable but Disengaged Profile), *OR* = 0.783, 95% CI [0.665, 0.921], *p* = 0.003; and Profile 5 (Highly Vulnerable and Disengaged Profile), *OR* = 0.553, 95% CI [0.410, 0.746], *p* < 0.001. Conversely, stronger incremental beliefs were associated with higher odds of belonging to Profile 3 rather than Profile 1 (Moderate Distress with Moderate Functioning Profile), *OR* = 1.545, 95% CI [1.282, 1.862], *p* < 0.001; Profile 2 (Psychologically Stable but Disengaged Profile), *OR* = 2.263, 95% CI [1.806, 2.835], *p* < 0.001; and Profile 5 (Highly Vulnerable and Disengaged Profile), *OR* = 2.452, 95% CI [1.487, 4.043], *p* < 0.001.

The comparison between Profile 3 and Profile 4 (Distressed but Engaged Profile) showed a different pattern. Stronger entity beliefs were associated with lower odds of belonging to Profile 3 rather than Profile 4, *OR* = 0.784, 95% CI [0.647, 0.951], *p* = 0.013. However, incremental beliefs did not significantly distinguish these two profiles, *OR* = 1.118, 95% CI [0.856, 1.460], *p* = 0.415. Thus, whereas both belief dimensions differentiated Profile 3 from Profiles 1, 2, and 5, only entity beliefs significantly differentiated it from Profile 4.

School connectedness consistently distinguished Profile 3 from all other profiles. Higher school connectedness was associated with higher odds of belonging to Profile 3 rather than Profile 1 (Moderate Distress with Moderate Functioning Profile), *OR* = 5.323, 95% CI [3.609, 7.852]; Profile 2 (Psychologically Stable but Disengaged Profile), *OR* = 5.638, 95% CI [3.699, 8.592]; Profile 4 (Distressed but Engaged Profile), *OR* = 7.365, 95% CI [4.671, 11.613]; and Profile 5 (Highly Vulnerable and Disengaged Profile), *OR* = 18.336, 95% CI [9.869, 34.069], all *p*s < 0.001.

Additional pairwise comparisons were conducted to characterize theoretically relevant differences between profiles. First, Profiles 4 (Distressed but Engaged Profile) and 5 (Highly Vulnerable and Disengaged Profile) were both characterized by elevated psychological distress but differed in academic engagement. Stronger incremental beliefs were associated with higher odds of belonging to Profile 4 rather than Profile 5, *OR* = 2.194, 95% CI [1.322, 3.641], *p* = 0.002. Higher school connectedness was also associated with higher odds of membership in Profile 4 rather than Profile 5, *OR* = 2.489, 95% CI [1.598, 3.879], *p* < 0.001. In contrast, stronger entity beliefs were associated with lower odds of belonging to Profile 4 rather than Profile 5, *OR* = 0.705, 95% CI [0.515, 0.964], *p* = 0.029.

Second, Profiles 2 (Psychologically Stable but Disengaged Profile) and 5 (Highly Vulnerable and Disengaged Profile) both showed low academic engagement but differed in mental well-being. Greater economic strain, *OR* = 0.579, 95% CI [0.353, 0.950], *p* = 0.030, and stronger entity beliefs, *OR* = 0.706, 95% CI [0.526, 0.949], *p* = 0.021, were associated with lower odds of belonging to Profile 2 rather than Profile 5. Conversely, higher school connectedness was associated with higher odds of belonging to Profile 2 rather than Profile 5, *OR* = 3.252, 95% CI [1.860, 5.688], *p* < 0.001. Demographic characteristics also distinguished these profiles. Older adolescents had lower odds of belonging to Profile 2 rather than Profile 5, *OR* = 0.726, 95% CI [0.536, 0.984], *p* = 0.039. Female adolescents likewise had lower odds than male adolescents of belonging to Profile 2 rather than Profile 5, *OR* = 0.407, 95% CI [0.177, 0.936], *p* = 0.034.

Third, Profiles 1 (Moderate Distress with Moderate Functioning Profile) and 4 (Distressed but Engaged Profile) were compared to examine characteristics associated with their contrasting levels of psychological distress. Higher paternal education was associated with higher odds of belonging to Profile 4 rather than Profile 1, *OR* = 3.648, 95% CI [1.787, 7.449], *p* < 0.001. Stronger incremental beliefs were also associated with higher odds of membership in Profile 4 rather than Profile 1, *OR* = 1.382, 95% CI [1.024, 1.867], *p* = 0.035. In contrast, higher school connectedness was associated with lower odds of belonging to Profile 4 rather than Profile 1, *OR* = 0.723, 95% CI [0.554, 0.944], *p* = 0.017.

Finally, family SSS did not significantly differentiate membership in any pair of profiles after adjustment for the other covariates and classroom clustering. In particular, its associations with membership in Profile 5 (Highly Vulnerable and Disengaged Profile) rather than Profile 1 (Moderate Distress with Moderate Functioning Profile), *OR* = 1.305, 95% CI [0.968, 1.759], *p* = 0.081, and Profile 5 rather than Profile 2 (Psychologically Stable but Disengaged Profile), *OR* = 1.351, 95% CI [0.991, 1.841], *p* = 0.057, were not statistically significant. These results provided no statistically significant evidence of an independent association between family SSS and profile membership in the adjusted model.

### 3.4. Perceived Academic Achievement as a Distal Outcome Across Latent Profiles

Perceived academic achievement was examined as a distal outcome using the BCH approach to account for classification uncertainty, with classroom clustering addressed through TYPE = MIXTURE COMPLEX. The analysis included 1830 adolescents with available achievement data. Following recommendations for distal outcome reporting (Carter, 2026), the Benjamini–Hochberg (BH) procedure was applied to control the false discovery rate at 0.05 across all 10 pairwise comparisons. The unadjusted and BH-adjusted *p*-values, standardized effect sizes, and corresponding 95% confidence intervals are presented in Table 4. The overall Wald test indicated significant differences in perceived academic achievement across the five profiles, χ^2^(4) = 125.136, *p* < 0.001. The corresponding global effect size was LTB-ω = 0.273, indicating a small overall association between latent profile membership and perceived academic achievement when interpreted on the metric of Cohen’s *d* (Lanza et al., 2013). Whereas the omnibus Wald test established that the profile-specific means were not all equal, LTB-ω summarized the overall magnitude of their separation across the five profiles.

Profile 3 (Flourishing and Engaged Profile) had the highest estimated mean achievement (*M* = 3.052, *SE* = 0.039), followed by Profile 4 (Distressed but Engaged Profile; *M* = 2.738, *SE* = 0.087) and Profile 1 (Moderate Distress with Moderate Functioning Profile; *M* = 2.628, *SE* = 0.059). Lower estimated means were observed for Profile 2 (Psychologically Stable but Disengaged Profile; *M* = 2.396, *SE* = 0.065) and Profile 5 (Highly Vulnerable and Disengaged Profile; *M* = 2.264, *SE* = 0.097).

Pairwise comparisons, evaluated using BH-adjusted *p*-values, indicated that Profile 3 had significantly higher perceived academic achievement than Profile 1, χ^2^(1) = 45.15, *p* < 0.001; Profile 2, χ^2^(1) = 58.36, *p* < 0.001; Profile 4, χ^2^(1) = 11.54, *p* = 0.001; and Profile 5, χ^2^(1) = 61.03, *p* < 0.001.

Among the remaining profiles, Profile 4 had significantly higher achievement than Profile 2, χ^2^(1) = 10.18, *p* = 0.002, and Profile 5, χ^2^(1) = 11.33, *p* = 0.001. Profile 1 also had significantly higher achievement than Profile 2, χ^2^(1) = 5.95, *p* = 0.018, and Profile 5, χ^2^(1) = 12.32, *p* = 0.001. No significant differences were observed between Profiles 1 and 4, χ^2^(1) = 1.34, *p* = 0.274, or between Profiles 2 and 5, χ^2^(1) = 1.07, *p* = 0.301.

## 4. Discussion

Informed by the PERMA framework and the dual-continua model, the present study aimed to extend previous research by integrating academic engagement with both positive and negative dimensions of mental well-being using a person-centered approach. Five distinct latent profiles were identified, demonstrating that adolescents’ academic engagement cannot be understood independently from their broader psychological functioning. Although a flourishing and engaged profile represented the most adaptive pattern, other configurations revealed that high academic engagement may coexist with severe psychological distress, whereas academic disengagement does not necessarily indicate elevated psychopathological symptoms. Furthermore, personal and contextual factors, particularly school connectedness and implicit theories of intelligence, differentiated adolescents across configurations, which in turn were associated with distinct levels of perceived academic achievement. Together, these findings provide a more nuanced understanding of adolescent developmental heterogeneity and underscore the critical importance of joint assessments across academic and psychological domains.

### 4.1. Diverse Configurations of Academic Engagement and Mental Well-Being

The present study extends previous person-centered research by jointly modeling academic engagement and both positive and negative dimensions of mental well-being, revealing configurations that may not be captured when these domains are examined separately. Previous studies have typically identified profiles based primarily on either mental well-being indicators and subsequently examined their associations with academic engagement, or derived engagement profiles and investigated their links with psychological outcomes (e.g., Sechague Monroy et al., 2024; M.-T. Wang & Peck, 2013). Although these studies have advanced our understanding of heterogeneity within specific domains, they may not fully capture how academic engagement and mental well-being co-occur within individuals. By integrating these dimensions into a unified profiling framework, the present study identified five distinct configurations, demonstrating that engagement and mental well-being can combine in diverse ways among adolescents.

The Flourishing and Engaged Profile and the Highly Vulnerable and Disengaged Profile represented two contrasting configurations. The former was characterized by high academic engagement, high positive mental well-being, and low psychological distress, whereas the latter showed the opposite pattern, with low academic engagement, reduced positive mental well-being, and elevated psychological distress. These profiles provided a clear contrast in overall functioning, whereas the other configurations revealed more nuanced combinations of academic engagement and mental well-being. Importantly, the identification of the Distressed but Engaged Profile revealed a more complex relationship between academic engagement and mental well-being. Although adolescents in this profile maintained relatively high academic engagement, they simultaneously experienced elevated psychological distress. This configuration suggests that academic engagement should not be regarded as an unequivocal indicator of adaptive functioning. Rather, high engagement may coexist with psychological difficulties, indicating that students’ involvement in academic activities needs to be interpreted alongside their broader mental well-being. This finding extends previous research on engaged-exhausted students (Salmela-Aro et al., 2016) by demonstrating that such a pattern can be identified when academic engagement is considered jointly with both positive and negative dimensions of mental well-being. Conversely, the Psychologically Stable but Disengaged Profile highlighted that low academic engagement does not necessarily reflect broader psychological maladjustment. Adolescents in this profile showed substantially lower academic engagement but maintained relatively favorable levels of mental well-being and lower psychological distress compared with more vulnerable profiles. This finding is consistent with the dual-continua model (Suldo & Doll, 2021), which proposes that positive mental well-being and psychopathological symptoms represent related but distinct dimensions. Thus, reduced academic engagement may reflect difficulties specific to academic motivation or involvement rather than a general deficit in mental well-being.

These findings extend previous variable-centered research demonstrating consistent associations between academic engagement and adolescent positive development (Suldo & Parker, 2022), by showing that these associations are not uniform across individuals. Rather than uniformly reflecting positive functioning, academic engagement may coexist with either positive well-being or psychological vulnerability across different profiles. Importantly, these configurations should not be considered universal patterns that necessarily emerge across all the samples. For example, although Salmela-Aro et al. (2016) identified an engaged-exhausted profile based on academic engagement and burnout indicators, this profile was not replicated in a subsequent study using comparable indicators (Yang et al., 2023). This suggests that the co-occurrence of high academic engagement and psychological difficulties may depend on developmental, contextual, or sample-specific characteristics (cf. Tamnes et al., 2025). Examining the joint configuration of academic engagement and mental well-being within specific populations is therefore essential for understanding adolescent heterogeneity.

### 4.2. Personal and Contextual Factors Associated with Diverse Academic Engagement–Mental Well-Being Configurations

Beyond identifying heterogeneous configurations of academic engagement and mental well-being, the present study examined personal and contextual characteristics associated with adolescents’ membership in these configurations. Overall, the findings indicated that engagement–well-being patterns were embedded within broader developmental contexts. Psychosocial resources showed the most consistent associations with profile membership, whereas demographic and socioeconomic characteristics demonstrated more selective patterns.

Among the examined psychosocial characteristics, school connectedness emerged as the most consistent correlate of profile membership, distinguishing the Flourishing and Engaged Profile (Profile 3) from every other profile. Its relevance also extended beyond comparisons with this optimal profile. Among adolescents experiencing psychological difficulties, higher school connectedness was associated with membership in the less distressed Moderate Distress with Moderate Functioning Profile (Profile 1) rather than the more Distressed but Engaged Profile (Profile 4). Between the two profiles characterized by elevated psychological distress, higher school connectedness was associated with membership in the Distressed but Engaged Profile (Profile 4) rather than the Highly Vulnerable and Disengaged Profile (Profile 5). Similarly, among adolescents with low academic engagement, higher school connectedness was associated with membership in the Psychologically Stable but Disengaged Profile (Profile 2) rather than the Highly Vulnerable and Disengaged Profile. Thus, school connectedness consistently distinguished profiles that retained a relatively favorable aspect of psychological or academic functioning from profiles characterized by broader vulnerability. These findings are consistent with ecological and developmental perspectives emphasizing schools as important social contexts associated with adolescents’ psychological adjustment and academic functioning (Allen et al., 2018; Wu et al., 2025). Feelings of belonging, social support, and emotional security at school may help explain why stronger school connectedness was associated with more favorable configurations of academic engagement and mental well-being (Gilbertson & Sanchez, 2026; Peng & Patterson, 2025). However, the cross-sectional nature of the analyses precludes conclusions about the direction of these associations.

Adolescents’ implicit theories of intelligence also showed differentiated associations with engagement–well-being configurations. Incremental beliefs were generally associated with comparatively more adaptive profiles, whereas stronger entity beliefs were associated with greater vulnerability. Both belief dimensions differentiated the Flourishing and Engaged Profile from Profiles 1, 2, and 5. However, only entity beliefs significantly distinguished the Flourishing and Engaged Profile from the Distressed but Engaged Profile (Profile 4), whereas incremental beliefs did not differentiate these two profiles. This differentiated pattern is consistent with a functional asymmetry between entity and incremental beliefs rather than their operation as simple opposites on a single continuum (Combette & Kelemen, 2025; Grüning et al., 2024; Scherer & Campos, 2022; Yan & Schuetze, 2023).

The additional comparisons further clarified this asymmetry. Among profiles involving psychological distress, stronger incremental beliefs were associated with membership in the Distressed but Engaged Profile (Profile 4) rather than with both the Highly Vulnerable and Disengaged Profile (Profile 5) and the Moderate Distress with Moderate Functioning Profile (Profile 1). Incremental beliefs were therefore associated with profiles characterized by the retention of academic engagement despite psychological difficulty. By contrast, stronger entity beliefs were associated with membership in the Highly Vulnerable and Disengaged Profile rather than the Distressed but Engaged Profile. They were also associated with membership in the Highly Vulnerable and Disengaged Profile rather than the Psychologically Stable but Disengaged Profile (Profile 2), suggesting that entity beliefs were more closely associated with psychological vulnerability even when the profiles shared similarly low academic engagement. Furthermore, stronger entity beliefs differentiated the Distressed but Engaged Profile from the Flourishing and Engaged Profile, indicating that fixed beliefs were associated with psychological distress even among adolescents who remained academically engaged. Collectively, these findings suggest that incremental beliefs may be particularly relevant to the coexistence of sustained academic engagement and psychological difficulty, whereas entity beliefs may be more consistently associated with vulnerable psychological functioning (Kapasi & Pei, 2022; Schleider et al., 2015; Yeager & Dweck, 2020). These beliefs may therefore represent potentially relevant intervention targets, although longitudinal and experimental research is required before causal or intervention conclusions can be drawn.

Beyond psychosocial resources, demographic and socioeconomic characteristics showed more selective associations with adolescents’ engagement–well-being configurations. Economic strain emerged as the most consistent socioeconomic correlate of psychological vulnerability. Greater economic strain was associated with higher odds of membership in the Moderate Distress with Moderate Functioning (Profile 1), Distressed but Engaged (Profile 4), and Highly Vulnerable and Disengaged Profiles (Profile 5) rather than the Flourishing and Engaged Profile (Profile 3). Economic strain did not distinguish the Psychologically Stable but Disengaged Profile (Profile 2) from the Flourishing and Engaged Profile, suggesting that its associations were more closely related to psychological difficulty than to low academic engagement alone. Consistent with this interpretation, greater economic strain was also associated with membership in the Highly Vulnerable and Disengaged Profile rather than the Psychologically Stable but Disengaged Profile, two profiles that shared low engagement but differed markedly in mental well-being. Economic strain may therefore represent a contextual risk factor associated with psychological vulnerability across profiles with different levels of academic engagement, consistent with the family stress model (Masarik & Conger, 2017).

Family-level socioeconomic indicators demonstrated less consistent patterns. Higher paternal education was associated with greater odds of membership in the Distressed but Engaged Profile (Profile 4) rather than both the Flourishing and Engaged Profile (Profile 3) and the Moderate Distress with Moderate Functioning Profile (Profile 1). This pattern indicates that greater paternal educational resources can coexist with sustained academic engagement and elevated psychological distress. One possible explanation is that higher parental education may be accompanied by stronger academic expectations or pressure in some families (Ma et al., 2018). However, academic expectations and pressure were not directly measured, so this interpretation remains tentative. Maternal education did not independently differentiate any profile from the Flourishing and Engaged Profile. Family SSS also did not significantly distinguish any pair of profiles after adjustment for the other covariates and classroom clustering. Maternal education and family SSS may overlap with other socioeconomic indicators or operate partly through economic strain, thereby weakening their unique associations with profile membership when these variables were entered simultaneously (Schisterman et al., 2009). Accordingly, the present findings provide no evidence that perceived family social standing was independently associated with profile membership.

Personal SSS showed a more consistent, although still selective, pattern than family SSS. Higher personal SSS was associated with greater odds of membership in the Flourishing and Engaged Profile (Profile 3) rather than the Moderate Distress with Moderate Functioning Profile (Profile 1), the Distressed but Engaged (Profile 4), and Highly Vulnerable and Disengaged Profiles (Profile 5). However, it did not differentiate the Psychologically Stable but Disengaged Profile (Profile 2) from the Flourishing and Engaged Profile. Taken together, personal SSS appeared more consistently associated with adolescents’ engagement–well-being configurations than family SSS, with its pattern of associations seeming more closely tied to psychological functioning than to academic engagement (Destin et al., 2012).

Demographic characteristics also demonstrated profile-specific associations. Associations involving sex did not follow a uniformly adaptive or maladaptive pattern. Female adolescents had lower odds than male adolescents of membership in the Psychologically Stable but Disengaged Profile (Profile 2) relative to the Flourishing and Engaged Profile (Profile 3) and, in a separate comparison, relative to the Highly Vulnerable and Disengaged Profile (Profile 5). Although seemingly divergent, this pattern is compatible with previous evidence that girls generally demonstrate stronger school engagement and scholastic performance while also showing greater vulnerability to internalizing symptoms during adolescence (Lam et al., 2012; Zahn-Waxler et al., 2008). Together, these contrasting tendencies are consistent with an integrative developmental perspective in which sex-linked biological, psychological, and social processes may have different implications across domains of functioning.

Age also showed selective associations with profile membership. Older adolescents had higher odds of belonging to the Distressed but Engaged Profile (Profile 4) and the Highly Vulnerable and Disengaged Profile (Profile 5) rather than the Flourishing and Engaged Profile (Profile 3). They also had higher odds of belonging to Profile 5 rather than the Psychologically Stable but Disengaged Profile (Profile 2). This pattern suggests that age was primarily associated with psychological vulnerability rather than academic disengagement alone and may reflect the increasing academic and developmental challenges encountered during adolescence. However, these associations should not be interpreted as evidence of developmental change with age. Students in Grades 7 and 8 were drawn from two junior high schools, whereas Grade 10 students attended a separate senior high school, and Grade 9 was not represented in the sample. Age was therefore intertwined with grade, cohort, and institutional context. Although the analyses accounted for classroom clustering, this adjustment addressed the nonindependence of observations rather than disentangling these overlapping characteristics. The observed associations may consequently reflect one or more of these contextual differences rather than age alone. Longitudinal research following students across continuous grade levels and multiple schools is needed to clarify whether the observed pattern reflects age-related developmental change.

Taken together, these findings extend the JD–R perspective (Bakker et al., 2014, 2023) by suggesting that the role of developmental resources and demands is not inherently determined but depends on the broader developmental contexts in which adolescents are embedded. Although psychosocial resources such as school connectedness and incremental beliefs were generally associated with more adaptive engagement–well-being configurations, their effects were shaped by the conditions under which adolescents experienced academic and developmental demands. For example, academic engagement could coexist with psychological difficulties when adolescents faced substantial pressures despite possessing certain adaptive resources. Family and demographic factors further illustrated this contextual complexity, as the same characteristics may function as either resources or demands depending on adolescents’ developmental circumstances. For instance, higher paternal education may provide academic resources while also being associated with heightened expectations and pressure, whereas socioeconomic conditions may represent either supportive contexts or sources of psychological strain. Similarly, demographic characteristics such as age and sex may reflect both developmental opportunities and vulnerabilities. Thus, the present findings suggest that the effects of resources and demands cannot be understood independently; rather, they should be considered within developmental contexts that shape whether academic engagement is accompanied by positive functioning or psychological vulnerability. However, the present study did not directly measure academic demands as conceptualized within the JD–R framework, nor did it examine resource–demand interactions, contextual moderation, or causal processes. Accordingly, the findings should be interpreted as conceptually consistent with, rather than as direct evidence for or a formal test of, the JD–R framework. In addition, because the R3STEP pairwise tests were exploratory and unadjusted for multiplicity, associations with *p*-values close to 0.05 should be interpreted cautiously.

### 4.3. Perceived Academic Achievement Across Diverse Academic Engagement–Mental Well-Being Configurations

Beyond identifying heterogeneous engagement–mental well-being configurations, the present study examined perceived academic achievement as a distal outcome to further characterize the identified profiles in relation to students’ perceptions of their academic performance. Perceived academic achievement differed significantly across the five profiles, with the highest level reported by adolescents in the Flourishing and Engaged Profile (Profile 3) and the lowest by those in the Highly Vulnerable and Disengaged Profile (Profile 5). Notably, although adolescents in the Distressed but Engaged Profile (Profile 4) maintained near-average academic engagement despite experiencing substantial psychological distress, their perceived academic achievement was significantly lower than that of Profile 3 and did not differ significantly from that of the Moderate Distress with Moderate Functioning Profile (Profile 1). This pattern suggests that maintaining academic engagement in the presence of substantial psychological distress may not necessarily be accompanied by correspondingly high perceived academic achievement. The lower perceived academic achievement of the Distressed but Engaged profile may reflect negatively biased self-evaluation, poorer academic functioning associated with distress-related concentration difficulties and reduced working-memory efficiency, or both (Owens et al., 2012; Platt et al., 2017). Because academic achievement was self-reported, the present study could not distinguish between these explanations. Taken together, academic engagement may be most adaptive when it co-occurs with positive psychological functioning. These findings extend previous research on academic engagement and achievement (Alrashidi et al., 2016; Salanova et al., 2010; Siu et al., 2014; Wong et al., 2024) by showing that perceived academic achievement differed across profiles characterized by distinct combinations of academic engagement and mental well-being. A different pattern emerged between the two profiles characterized by low academic engagement. Although the Psychologically Stable but Disengaged Profile (Profile 2) and the Highly Vulnerable and Disengaged Profile (Profile 5) differed markedly in mental well-being, they exhibited comparably low academic engagement and did not differ significantly in perceived academic achievement. Thus, within this particular comparison, substantial differences in mental well-being were not accompanied by statistically distinguishable differences in perceived academic achievement.

Taken together, these findings extend previous research by showing that perceived academic achievement is not simply a function of adolescents’ academic engagement or mental well-being alone (Schnitzler et al., 2021; Sechague Monroy et al., 2024; Suldo & Shaffer, 2008; M.-T. Wang & Peck, 2013); rather, it depends on the specific configurations of these two domains. These findings showed that distinct engagement–well-being configurations were accompanied by differences in how adolescents perceived their academic performance, highlighting the importance of interpreting academic engagement within the broader context of mental well-being. Nevertheless, because perceived academic achievement was assessed concurrently using a brief self-report measure with modest internal consistency, these findings should not be interpreted as establishing the external validity of the profiles or as demonstrating differences in objectively measured academic achievement.

### 4.4. Theoretical and Practical Implications

The present findings can be situated within three complementary theoretical perspectives. First, the heterogeneous configurations of academic engagement and positive mental well-being are compatible with the PERMA framework, which treats engagement, positive emotion, and meaning as related but distinguishable elements of well-being. Although academic engagement is more domain-specific than engagement as conceptualized in PERMA, the identified profiles indicate that engagement and other aspects of positive functioning do not necessarily vary in parallel. Second, the distinct combinations of positive mental well-being and psychopathological symptoms are consistent with the dual-continua model of mental health, which conceptualizes these dimensions as related but not as opposite ends of a single continuum. The profiles further illustrate that both dimensions can combine differently with academic engagement. Third, the differentiated associations of psychosocial and socioeconomic characteristics with profile membership can be interpreted within the JD–R perspective, which emphasizes the relevance of resources and demands to engagement and well-being.

From a practical perspective, the findings highlight the importance of promoting adolescents’ academic engagement within a broader framework of mental well-being. Consistent with the goals of positive education (Seligman et al., 2009), schools should move beyond a narrow focus on academic achievement and engagement by simultaneously fostering positive psychological functioning. The identification of heterogeneous engagement–mental well-being configurations suggests that increasing academic engagement alone may not be sufficient, as some adolescents may remain highly engaged while experiencing substantial psychological difficulties. Instead, educational practices should aim to cultivate adaptive combinations of engagement and mental well-being. The present findings draw attention to psychosocial factors associated with profile membership. School connectedness and implicit theories of intelligence may offer two candidate directions for future intervention research (Attri et al., 2025; Canning & Limeri, 2023). Given the complex and profile-specific associations of personal and contextual characteristics with profile membership, these focused components may be best embedded within more comprehensive school-based programs addressing social-emotional functioning, connectedness, and academic development, such as school counselor–delivered interventions that integrate social-emotional and academic support (Lemberger et al., 2015, 2018; Lemberger-Truelove et al., 2025). Accordingly, schools may consider incorporating dual-factor screening into tiered support systems by assessing psychological distress and positive functioning alongside academic engagement. School counselors, psychologists, and social workers can then follow up with students reporting elevated distress despite comparatively preserved engagement and coordinate targeted support or referral when needed.

### 4.5. Limitations and Future Directions

Several limitations of the present study should be acknowledged. First, the cross-sectional design limits conclusions regarding the temporal ordering and causal relationships among academic engagement, mental well-being, and associated factors. Future longitudinal studies using latent transition analysis are needed to examine the stability and developmental changes in engagement–well-being configurations over time.

Second, the generalizability of the findings is limited by the use of a convenience sample, which resulted in incomplete coverage of grade levels and a restricted range of institutional contexts. The small number of schools also precluded a meaningful examination of school-level heterogeneity. In addition, given the limited methodological guidance on profile enumeration with nested data, classroom clustering was not incorporated into the primary profile-enumeration models. Future studies should recruit larger multilevel samples that include a more complete range of grade levels and more classrooms across a broader range of schools and regions, thereby enabling multilevel LPA and examination of the robustness and generalizability of the identified profile structure across educational contexts.

Third, the variables associated with profile membership were modeled primarily as additive main effects, and the study did not directly assess a comprehensive set of academic and contextual demands. Future research with larger samples could use flexible analytic approaches, including machine-learning methods, to explore potentially nonlinear and interactive associations among developmental resources, directly measured demands, and engagement–well-being configurations.

Fourth, academic engagement was assessed using a single composite score, although its behavioral, emotional, cognitive, and agentic components may show distinct associations with mental well-being and academic outcomes. Future studies should therefore incorporate multidimensional measures of engagement. In addition, because all profile indicators, associated characteristics, and perceived academic achievement relied exclusively on concurrent adolescent self-reports, the observed associations may be subject to shared-method variance and common response tendencies. Although self-reports are well-suited for capturing subjective psychological experiences, future research should incorporate objective indicators (e.g., school grades and standardized achievement tests) and multi-informant assessments (e.g., parent or teacher reports) to determine whether these profile differences hold across diverse data sources.

## 5. Conclusions

The present study advances understanding of adolescent development by demonstrating that academic engagement and mental well-being represent distinct yet related domains that can form heterogeneous configurations. Consistent with the PERMA framework and the dual-continua model, the findings show that academic engagement does not necessarily indicate flourishing, as engagement may coexist with psychological vulnerability. Furthermore, the differential associations of personal, socioeconomic, and psychosocial characteristics with profile membership were broadly consistent with the JD–R perspective. Overall, promoting adolescent positive development requires approaches that foster the co-occurrence of academic engagement and mental well-being.

## Figures and Tables

**Table 1:** Descriptive Statistics and Correlations of Latent Profile Indicators, Covariates, and Distal Outcome.

Variable		*M*	*SD*	1	2	3	4	5	6	7	8	9	10	11	12	13	14	15	16	17	18
1.	Anxiety	1.64	0.40	—																	
2.	Depression	1.64	0.50	0.77 ***	—																
3.	Negative affect	2.12	0.77	0.59 ***	0.60 ***	—															
4.	Life satisfaction	4.54	1.44	−0.48 ***	−0.57 ***	−0.38 ***	—														
5.	Positive affect	3.21	0.87	−0.34 ***	−0.44 ***	−0.12 ***	0.45 ***	—													
6.	Meaning in life	4.18	1.53	−0.34 ***	−0.41 ***	−0.27 ***	0.41 ***	0.47 ***	—												
7.	Academic engagement	4.51	1.50	−0.42 ***	−0.50 ***	−0.31 ***	0.52 ***	0.51 ***	0.50 ***	—											
8.	Female	0.45	0.50	0.15 ***	0.12 ***	0.15 ***	−0.07 **	−0.09 ***	−0.06 **	0.00	—										
9.	Age	14.39	1.41	0.20 ***	0.21 ***	0.18 ***	−0.17 ***	−0.09 ***	−0.11 ***	−0.14 ***	−0.03	—									
10.	Father education	0.26	0.44	−0.05 *	−0.03	0.00	0.09 ***	0.12 ***	0.09 ***	0.10 ***	0.01	−0.13 ***	—								
11.	Mother education	0.22	0.42	−0.04	−0.02	0.00	0.06 *	0.09 ***	0.06 *	0.08 ***	0.02	−0.14 ***	0.62 ***	—							
12.	Economic strain	1.29	0.58	0.25 ***	0.28 ***	0.19 ***	−0.34 ***	−0.18 ***	−0.18 ***	−0.16 ***	0.03	0.20 ***	−0.12 ***	−0.14 ***	—						
13.	Family SSS	5.40	1.44	−0.15 ***	−0.21 ***	−0.16 ***	0.30 ***	0.22 ***	0.23 ***	0.20 ***	−0.06 *	−0.21 ***	0.23 ***	0.20 ***	−0.34 ***	—					
14.	Personal SSS	5.65	1.63	−0.19 ***	−0.26 ***	−0.14 ***	0.21 ***	0.31 ***	0.23 ***	0.25 ***	−0.04	0.00	0.11 ***	0.08 ***	−0.09 ***	0.42 ***	—				
15.	Entity belief	2.80	1.40	0.30 ***	0.37 ***	0.28 ***	−0.32 ***	−0.31 ***	−0.34 ***	−0.40 ***	0.08 ***	0.16 ***	−0.02	0.00	0.17 ***	−0.17 ***	−0.16 ***	—			
16.	Incremental belief	3.41	1.22	−0.26 ***	−0.28 ***	−0.19 ***	0.34 ***	0.36 ***	0.37 ***	0.42 ***	−0.04	−0.05 *	0.06 **	0.02	−0.14 ***	0.14 ***	0.15 ***	−0.40 ***	—		
17.	School connectedness	3.88	0.88	−0.47 ***	−0.57 ***	−0.40 ***	0.50 ***	0.47 ***	0.38 ***	0.55 ***	−0.09 ***	−0.20 ***	0.07 **	0.07 **	−0.22 ***	0.21 ***	0.27 ***	−0.34 ***	0.30 ***	—	
18.	Perceived academic achievement	2.82	0.79	−0.20 ***	−0.26 ***	−0.17 ***	0.26 ***	0.33 ***	0.25 ***	0.39 ***	0.06 **	−0.11 ***	0.19 ***	0.17 ***	−0.10 ***	0.22 ***	0.37 ***	−0.18 ***	0.21 ***	0.26 ***	—

*Note.* Female was dummy coded as 0 = boys and 1 = girls. Father’s education and mother’s education were dummy coded as 0 = no higher education and 1 = higher education. * *p* < 0.05, ** *p* < 0.01, *** *p* < 0.001.

**Table 2:** Fit Indices for Latent Profile Analysis Models.

Model & Profiles	Par	LL	AIC	CAIC	BIC	aBIC	AWE	Entropy	VLMR *p*	aLMR *p*	BLRT *p*	Min Class Size (*n*, %)
**Model 1**												
1 Profile	14	−18,319.252	36,666.505	36,757.810	36,743.811	36,699.333	36,891.116	—	—	—	—	1848 (100.00%)
2 Profiles	22	−16,604.602	33,253.204	33,396.685	33,374.685	33,304.791	33,606.166	0.849	<0.001	<0.001	<0.001	671 (36.31%)
3 Profiles	30	−15,917.534	31,895.068	32,090.724	32,060.724	31,965.415	32,376.380	0.838	<0.001	<0.001	<0.001	347 (18.78%)
4 Profiles	38	−15,699.805	31,475.611	31,723.441	31,685.442	31,564.717	32,085.271	0.816	0.055	0.057	<0.001	189 (10.23%)
5 Profiles	46	−15,541.615	31,175.230	31,475.236	31,429.236	31,283.095	31,913.241	0.799	0.010	0.011	<0.001	179 (9.69%)
6 Profiles	54	−15,444.492	30,996.983	31,349.164	31,295.163	31,123.607	31,863.345	0.775	0.257	0.261	<0.001	122 (6.60%)
**Model 2**												
1 Profile	14	−18,319.252	36,666.505	36,757.810	36,743.811	36,699.333	36,891.116	—	—	—	—	1848 (100.00%)
2 Profiles	29	−16,312.883	32,683.767	32,872.900	32,843.900	32,751.768	33,149.034	0.841	<0.001	<0.001	<0.001	799 (43.24%)
3 Profiles	44	−15,631.051	31,350.102	31,637.064	31,593.064	31,453.277	32,056.026	0.839	0.003	0.003	<0.001	406 (21.97%)
4 Profiles	59	−15,341.884	30,801.769	31,186.558	31,127.558	30,940.117	31,748.347	0.846	<0.001	<0.001	<0.001	140 (7.58%)
5 Profiles	74	−15,176.449	30,500.897	30,983.516	30,909.515	30,674.419	31,688.133	0.800	0.008	0.009	<0.001	142 (7.68%)
6 Profiles	89	−15,046.089	30,270.178	30,850.623	30,761.624	30,478.873	31,698.069	0.798	0.334	0.338	<0.001	110 (5.95%)
**Model 3**												
1 Profile	35	−15,477.446	31,024.892	31,253.157	31,218.157	31,106.963	31,586.422	—	—	—	—	1848 (100.00%)
2 Profiles	43	−15,274.062	30,634.124	30,914.564	30,871.564	30,734.954	31,324.004	0.837	<0.001	<0.001	<0.001	358 (19.37%)
3 Profiles	51	−15,198.747	30,499.494	30,832.109	30,781.109	30,619.084	31,317.724	0.814	<0.001	<0.001	<0.001	196 (10.61%)
4 Profiles	59	−15,117.111	30,352.223	30,737.012	30,678.012	30,490.571	31,298.801	0.843	0.002	0.002	<0.001	141 (7.63%)
**5 Profiles**	**67**	**−15,061.965**	**30,257.931**	**30,694.895**	**30,627.896**	**30,415.038**	**31,332.859**	**0.** **811**	**<0.001**	**0.001**	**<0.001**	**98 (5.30%)**
6 Profiles	75	−15,022.460	30,194.919	30,684.059	30,609.059	30,370.786	31,398.199	0.819	0.032	0.034	<0.001	25 (1.35%)
**Model 4**												
1 Profile	35	−15,477.446	31,024.892	31,253.157	31,218.157	31,106.963	31,586.422	—	—	—	—	1848 (100.00%)
2 Profiles	50	−15,161.307	30,422.614	30,748.707	30,698.707	30,539.858	31,224.800	0.592	<0.001	<0.001	<0.001	873 (47.24%)
3 Profiles	65	−14,968.567	30,067.135	30,491.055	30,426.055	30,219.552	31,109.976	0.679	<0.001	<0.001	<0.001	377 (20.40%)
4 Profiles	80	−14,888.099	29,936.198	30,457.947	30,377.946	30,123.789	31,219.695	0.688	0.376	0.380	<0.001	137 (7.41%)
5 Profiles	95	−14,806.068	29,802.137	30,421.713	30,326.713	30,024.901	31,326.289	0.660	0.060	0.062	<0.001	110 (5.95%)
6 Profiles	110	−14,740.540	29,701.081	30,418.485	30,308.485	29,959.018	31,465.889	0.752	0.478	0.481	<0.001	99 (5.36%)
**Model 5**												
1 Profile	35	−15,477.446	31,024.892	31,253.157	31,218.157	31,106.963	31,586.422	—	—	—	—	1848 (100.00%)
2 Profiles	64	−15,140.609	30,409.218	30,826.617	30,762.617	30,559.290	31,436.016	0.520	<0.001	<0.001	<0.001	828 (44.81%)
3 Profiles	93	−14,978.297	30,142.594	30,749.127	30,656.127	30,360.668	31,634.660	0.637	<0.001	<0.001	<0.001	335 (18.13%)
4 Profiles	122	−14,891.718	30,027.436	30,823.103	30,701.103	30,313.512	31,984.770	0.685	0.031	0.032	<0.001	69 (3.73%)
5 Profiles	151	−14,810.573	29,923.145	30,907.947	30,756.946	30,277.223	32,345.747	0.774	0.598	0.599	<0.001	74 (4.00%)
6 Profiles	180	−14,743.602	29,847.204	31,021.139	30,841.138	30,269.283	32,735.073	0.671	0.227	0.227	<0.001	78 (4.22%)
**Model 6**												
1 Profile	35	−15,477.446	31,024.892	31,253.157	31,218.157	31,106.963	31,586.422	—	—	—	—	1848 (100.00%)
2 Profiles	71	−14,947.622	30,037.244	30,500.296	30,429.296	30,203.731	31,176.348	0.670	<0.001	<0.001	<0.001	787 (42.59%)
3 Profiles	107	−14,792.739	29,799.479	30,497.317	30,390.318	30,050.382	31,516.156	0.741	0.573	0.574	<0.001	171 (9.25%)
4 Profiles	143	−14,692.844	29,671.689	30,604.314	30,461.315	30,007.008	31,965.940	0.775	0.183	0.183	<0.001	122 (6.60%)
5 Profiles	179	−14,598.432	29,554.865	30,722.277	30,543.278	29,974.600	32,426.690	0.778	0.085	0.086	<0.001	106 (5.74%)
6 Profiles	215	−14,515.477	29,460.954	30,863.154	30,648.154	29,965.105	32,910.353	0.701	0.322	0.322	<0.001	56 (3.03%)

*Note.* Par = number of free parameters; LL = model log-likelihood; AIC = Akaike information criterion; CAIC = consistent Akaike information criterion; BIC = Bayesian information criterion; aBIC = sample-size-adjusted Bayesian information criterion; AWE = approximate weight of evidence criterion; Entropy indicates overall classification precision. VLMR = Vuong–Lo–Mendell–Rubin likelihood ratio test; aLMR = adjusted Lo–Mendell–Rubin likelihood ratio test; BLRT = bootstrapped likelihood ratio test. The VLMR, aLMR, and BLRT columns report *p*-values. Min Class Size = number and percentage of participants in the smallest profile based on most likely class membership.

**Table 3:** Model Classification Diagnostics.

Profile	Proportions	95% CI	mcaP*_k_*	AvePP*_k_*	OCC*_k_*
Profile 1	21.71%	[7.50%, 26.00%]	21.37%	0.79	13.33
Profile 2	10.64%	[8.30%, 12.90%]	10.17%	0.88	63.04
Profile 3	55.27%	[50.80%, 67.10%]	56.22%	0.92	9.30
Profile 4	6.88%	[4.60%, 9.10%]	6.93%	0.86	84.28
Profile 5	5.50%	[3.80%, 8.00%]	5.30%	0.86	106.43

*Note.* Proportions = Model-estimated latent class proportions based on estimated class probabilities; mcaP*_k_* = Modal class assignment proportion (percentage of individuals assigned to each class based on most likely class membership); AvePP*_k_* = Average posterior class probabilities; OCC*_k_* = Odds of correct classification.

**Table 4:** Pairwise Comparisons of Perceived Academic Achievement Across Latent Profiles.

Comparison	Mean Difference	Unadjusted *p*	BH-Adjusted *p*	Cohen’s *d*	95% CI for *d*
Profile 1 vs. Profile 2	0.232	0.015	0.018	0.340	[0.168, 0.512]
Profile 1 vs. Profile 3	−0.424	<0.001	<0.001	−0.579	[−0.697, −0.461]
Profile 1 vs. Profile 4	−0.110	0.246	0.274	−0.142	[−0.343, 0.058]
Profile 1 vs. Profile 5	0.364	<0.001	0.001	0.481	[0.261, 0.702]
Profile 2 vs. Profile 3	−0.656	<0.001	<0.001	−0.943	[−1.101, −0.786]
Profile 2 vs. Profile 4	−0.342	0.001	0.002	−0.463	[−0.690, −0.236]
Profile 2 vs. Profile 5	0.132	0.301	0.301	0.183	[−0.058, 0.424]
Profile 3 vs. Profile 4	0.314	<0.001	0.001	0.400	[0.214, 0.586]
Profile 3 vs. Profile 5	0.788	<0.001	<0.001	1.026	[0.817, 1.235]
Profile 4 vs. Profile 5	0.474	<0.001	0.001	0.587	[0.319, 0.855]

*Note.* Mean differences were calculated as the first profile minus the second profile. Unadjusted *p*-values are from BCH pairwise Wald tests; BH-adjusted *p*-values were obtained by applying the Benjamini–Hochberg procedure across the 10 pairwise comparisons; Cohen’s *d* and its 95% confidence intervals were calculated from class-specific variances and effective class sizes.

## Data Availability

The data are not publicly available due to privacy and ethical restrictions involving minors and children. De-identified data may be made available from the corresponding author upon reasonable request and subject to approval by the relevant ethics committee and administrative authorities.

## Abbreviations

The following abbreviations are used in this manuscript:

aBIC	sample-size adjusted Bayesian information criterion
AIC	Akaike information criterion
aLMR	Lo-Mendell-Rubin adjusted likelihood ratio test
AvePP*_k_*	Average posterior class probabilities
AWE	Approximate weight of evidence criterion
BH	Benjamini–Hochberg procedure
BIC	Bayesian information criterion
BLRT	Bootstrapped likelihood ratio test
CAIC	Consistent Akaike information criterion
CI	Confidence interval
FIML	Full information maximum likelihood
JD–R	Job demands–resources model
LPA	Latent profile analysis
MAR	Missing at random
mcaP*_k_*	Modal class assignment proportion (percentage of individuals assigned to each class based on most likely class membership)
MCAR	Missing completely at random
MLR	Robust maximum likelihood
OCC*_k_*	Odds of correct classification
OR	Odds ratio
PERMA	Positive emotion, engagement, relationships, meaning and accomplishment
SSS	Subjective social status
VLMR	Vuong-Lo-Mendell-Rubin likelihood ratio test

## Appendix A

**Table A1:** Pairwise Comparisons of Model-Estimated Indicator Means Across the Five Profiles.

Indicator	Contrast (i − j)	*M_i_*	*M_j_*	Mean Difference	*SE*	95% Bootstrap CI	*p*
**Anxiety**	1 − 2	0.531	−0.086	0.617	0.279	[0.402, 1.673]	0.027
	1 − 3	0.531	−0.502	1.033	0.249	[0.848, 2.007]	<0.001
	1 − 4	0.531	1.463	−0.932	0.392	[−1.246, 0.540]	0.018
	1 − 5	0.531	1.276	−0.745	0.399	[−1.127, 0.694]	0.061
	2 − 3	−0.086	−0.502	0.415	0.074	[0.247, 0.538]	<0.001
	2 − 4	−0.086	1.463	−1.549	0.181	[−1.817, −1.114]	<0.001
	2 − 5	−0.086	1.276	−1.363	0.189	[−1.696, −0.931]	<0.001
	3 − 4	−0.502	1.463	−1.964	0.185	[−2.206, −1.452]	<0.001
	3 − 5	−0.502	1.276	−1.778	0.206	[−2.114, −1.263]	<0.001
	4 − 5	1.463	1.276	0.186	0.227	[−0.265, 0.584]	0.413
**Depression**	1 − 2	0.642	−0.198	0.840	0.160	[0.664, 1.354]	<0.001
	1 − 3	0.642	−0.638	1.280	0.106	[1.159, 1.620]	<0.001
	1 − 4	0.642	1.879	−1.237	0.189	[−1.393, −0.584]	<0.001
	1 − 5	0.642	1.906	−1.264	0.260	[−1.508, −0.398]	<0.001
	2 − 3	−0.198	−0.638	0.440	0.087	[0.208, 0.567]	<0.001
	2 − 4	−0.198	1.879	−2.077	0.118	[−2.289, −1.823]	<0.001
	2 − 5	−0.198	1.906	−2.104	0.166	[−2.398, −1.701]	<0.001
	3 − 4	−0.638	1.879	−2.517	0.130	[−2.684, −2.097]	<0.001
	3 − 5	−0.638	1.906	−2.544	0.195	[−2.777, −1.940]	<0.001
	4 − 5	1.879	1.906	−0.027	0.172	[−0.328, 0.355]	0.877
**Negative affect**	1 − 2	0.516	−0.126	0.642	0.267	[0.395, 1.559]	0.016
	1 − 3	0.516	−0.389	0.905	0.235	[0.707, 1.734]	<0.001
	1 − 4	0.516	0.978	−0.462	0.373	[−0.824, 0.850]	0.215
	1 − 5	0.516	0.893	−0.377	0.375	[−0.778, 0.870]	0.315
	2 − 3	−0.126	−0.389	0.263	0.083	[0.094, 0.415]	0.001
	2 − 4	−0.126	0.978	−1.104	0.188	[−1.440, −0.681]	<0.001
	2 − 5	−0.126	0.893	−1.019	0.196	[−1.387, −0.607]	<0.001
	3 − 4	−0.389	0.978	−1.367	0.188	[−1.670, −0.894]	<0.001
	3 − 5	−0.389	0.893	−1.282	0.208	[−1.658, −0.798]	<0.001
	4 − 5	0.978	0.893	0.085	0.235	[−0.400, 0.523]	0.719
**Life satisfaction**	1 − 2	−0.476	−0.262	−0.214	0.124	[−0.468, 0.019]	0.084
	1 − 3	−0.476	0.429	−0.905	0.090	[−1.064, −0.722]	<0.001
	1 − 4	−0.476	−0.756	0.280	0.159	[−0.122, 0.530]	0.077
	1 − 5	−0.476	−0.980	0.504	0.151	[0.126, 0.731]	0.001
	2 − 3	−0.262	0.429	−0.692	0.098	[−0.874, −0.484]	<0.001
	2 − 4	−0.262	−0.756	0.494	0.150	[0.175, 0.774]	0.001
	2 − 5	−0.262	−0.980	0.718	0.145	[0.389, 0.970]	<0.001
	3 − 4	0.429	−0.756	1.186	0.139	[0.843, 1.403]	<0.001
	3 − 5	0.429	−0.980	1.410	0.139	[1.055, 1.619]	<0.001
	4 − 5	−0.756	−0.980	0.224	0.156	[−0.095, 0.535]	0.150
**Positive affect**	1 − 2	−0.226	−0.267	0.040	0.130	[−0.191, 0.315]	0.756
	1 − 3	−0.226	0.291	−0.517	0.128	[−0.701, −0.151]	<0.001
	1 − 4	−0.226	−0.462	0.236	0.155	[−0.032, 0.568]	0.127
	1 − 5	−0.226	−0.944	0.718	0.137	[0.429, 0.973]	<0.001
	2 − 3	−0.267	0.291	−0.558	0.110	[−0.742, −0.322]	<0.001
	2 − 4	−0.267	−0.462	0.195	0.154	[−0.085, 0.506]	0.205
	2 − 5	−0.267	−0.944	0.677	0.160	[0.332, 0.960]	<0.001
	3 − 4	0.291	−0.462	0.753	0.125	[0.488, 0.991]	<0.001
	3 − 5	0.291	−0.944	1.235	0.147	[0.849, 1.463]	<0.001
	4 − 5	−0.462	−0.944	0.482	0.170	[0.114, 0.777]	0.005
**Meaning in life**	1 − 2	−0.277	−0.343	0.066	0.133	[−0.192, 0.334]	0.620
	1 − 3	−0.277	0.313	−0.590	0.104	[−0.767, −0.403]	<0.001
	1 − 4	−0.277	−0.381	0.103	0.168	[−0.236, 0.391]	0.538
	1 − 5	−0.277	−0.908	0.631	0.159	[0.252, 0.887]	<0.001
	2 − 3	−0.343	0.313	−0.656	0.098	[−0.844, −0.450]	<0.001
	2 − 4	−0.343	−0.381	0.038	0.167	[−0.290, 0.355]	0.821
	2 − 5	−0.343	−0.908	0.565	0.152	[0.230, 0.820]	<0.001
	3 − 4	0.313	−0.381	0.693	0.145	[0.405, 0.936]	<0.001
	3 − 5	0.313	−0.908	1.221	0.146	[0.870, 1.451]	<0.001
	4 − 5	−0.381	−0.908	0.528	0.189	[0.121, 0.859]	0.005
**Academic engagement**	1 − 2	−0.218	−1.493	1.275	0.114	[1.057, 1.451]	<0.001
	1 − 3	−0.218	0.536	−0.754	0.114	[−0.942, −0.508]	<0.001
	1 − 4	−0.218	0.083	−0.302	0.147	[−0.616, −0.055]	0.040
	1 − 5	−0.218	−1.731	1.513	0.142	[1.086, 1.678]	<0.001
	2 − 3	−1.493	0.536	−2.029	0.076	[−2.160, −1.855]	<0.001
	2 − 4	−1.493	0.083	−1.576	0.117	[−1.811, −1.357]	<0.001
	2 − 5	−1.493	−1.731	0.239	0.132	[−0.090, 0.441]	0.070
	3 − 4	0.536	0.083	0.453	0.124	[0.143, 0.640]	<0.001
	3 − 5	0.536	−1.731	2.267	0.140	[1.837, 2.425]	<0.001
	4 − 5	0.083	−1.731	1.815	0.128	[1.542, 2.008]	<0.001

*Note.* N = 1848. Profile numbering corresponds to Figure 1. For each contrast i − j, *M_i_* and *M_j_* are the model-estimated indicator means for the first- and second-listed profiles, respectively. The mean difference is *M_i_* − *M_j_*; positive values indicate a higher mean in the first-listed profile. Means and differences are expressed in z-score units. Mean differences were taken directly from the model output and may differ slightly from differences calculated using the rounded means. *SE* = bootstrap standard error; CI = confidence interval. Estimates were obtained using ML, with 1000 completed bootstrap resamples. The 95% percentile bootstrap CIs use the Lower 2.5% and Upper 2.5% limits in the Mplus output. *p*-values are the two-sided normal-approximation values reported by Mplus, based on the estimate-to-SE ratio, and may not agree with the percentile bootstrap CIs. All comparisons and CIs are unadjusted for multiple comparisons.

**Table A2:** Covariates Logits and Odds Ratios for the Five-Profile Model.

Profile Membership	Reference Profile									
	1		2		3		4		5	
	*Logit B*	*OR*	*Logit B*	*OR*	*Logit B*	*OR*	*Logit B*	*OR*	*Logit B*	*OR*
**Female**										
1. Moderate Distress with Moderate Functioning	—	—	1.246 ***	3.476	0.139	1.150	−0.127	0.881	0.346	1.413
2. Psychologically Stable but Disengaged	−1.246 ***	0.288	—	—	−1.107 ***	0.331	−1.373 ***	0.253	−0.900 *	0.407
3. Flourishing and Engaged	−0.139	0.870	1.107 ***	3.024	—	—	−0.267	0.766	0.207	1.230
4. Distressed but Engaged	0.127	1.136	1.373 ***	3.948	0.267	1.305	—	—	0.473	1.605
5. Highly Vulnerable and Disengaged	−0.346	0.707	0.900 *	2.460	−0.207	0.813	−0.473	0.623	—	—
**Age**										
1. Moderate Distress with Moderate Functioning	—	—	0.180	1.198	0.145	1.156	−0.082	0.921	−0.140	0.870
2. Psychologically Stable but Disengaged	−0.180	0.835	—	—	−0.036	0.965	−0.263	0.769	−0.320 *	0.726
3. Flourishing and Engaged	−0.145	0.865	0.036	1.036	—	—	−0.227 *	0.797	−0.284 *	0.753
4. Distressed but Engaged	0.082	1.086	0.263	1.301	0.227 *	1.255	—	—	−0.057	0.945
5. Highly Vulnerable and Disengaged	0.140	1.150	0.320 *	1.377	0.284 *	1.329	0.057	1.059	—	—
**Father’s Education**										
1. Moderate Distress with Moderate Functioning	—	—	−0.690	0.501	−0.551	0.576	−1.294 ***	0.274	−0.695	0.499
2. Psychologically Stable but Disengaged	0.690	1.994	—	—	0.139	1.149	−0.604	0.547	−0.005	0.995
3. Flourishing and Engaged	0.551	1.735	−0.139	0.870	—	—	−0.743 *	0.476	−0.144	0.866
4. Distressed but Engaged	1.294 ***	3.648	0.604	1.829	0.743 *	2.102	—	—	0.599	1.820
5. Highly Vulnerable and Disengaged	0.695	2.005	0.005	1.005	0.144	1.155	−0.599	0.549	—	—
**Mother’s Education**										
1. Moderate Distress with Moderate Functioning	—	—	0.412	1.510	0.629	1.876	0.341	1.406	1.230	3.421
2. Psychologically Stable but Disengaged	−0.412	0.662	—	—	0.217	1.242	−0.072	0.931	0.818	2.265
3. Flourishing and Engaged	−0.629	0.533	−0.217	0.805	—	—	−0.288	0.749	0.601	1.823
4. Distressed but Engaged	−0.341	0.711	0.072	1.074	0.288	1.334	—	—	0.889	2.433
5. Highly Vulnerable and Disengaged	−1.230	0.292	−0.818	0.442	−0.601	0.548	−0.889	0.411	—	—
**Economic Strain**										
1. Moderate Distress with Moderate Functioning	—	—	0.560 *	1.750	0.631 **	1.880	−0.272	0.762	0.013	1.013
2. Psychologically Stable but Disengaged	−0.560 *	0.571	—	—	0.072	1.074	−0.831 **	0.436	−0.546 *	0.579
3. Flourishing and Engaged	−0.631 **	0.532	−0.072	0.931	—	—	−0.903 ***	0.405	−0.618 *	0.539
4. Distressed but Engaged	0.272	1.312	0.831 **	2.296	0.903 ***	2.467	—	—	0.285	1.329
5. Highly Vulnerable and Disengaged	−0.013	0.987	0.546 *	1.727	0.618 *	1.855	−0.285	0.752	—	—
**Family Subjective Social Status**										
1. Moderate Distress with Moderate Functioning	—	—	0.035	1.035	−0.120	0.887	0.012	1.012	−0.266	0.766
2. Psychologically Stable but Disengaged	−0.035	0.966	—	—	−0.155	0.857	−0.023	0.977	−0.301	0.740
3. Flourishing and Engaged	0.120	1.128	0.155	1.167	—	—	0.132	1.141	−0.146	0.864
4. Distressed but Engaged	−0.012	0.988	0.023	1.023	−0.132	0.876	—	—	−0.278	0.758
5. Highly Vulnerable and Disengaged	0.266	1.305	0.301	1.351	0.146	1.157	0.278	1.320	—	—
**Personal Subjective Social Status**										
1. Moderate Distress with Moderate Functioning	—	—	−0.040	0.961	−0.152 *	0.859	0.113	1.120	0.092	1.096
2. Psychologically Stable but Disengaged	0.040	1.040	—	—	−0.112	0.894	0.153	1.165	0.132	1.141
3. Flourishing and Engaged	0.152 *	1.164	0.112	1.119	—	—	0.265 *	1.304	0.244 *	1.276
4. Distressed but Engaged	−0.113	0.893	−0.153	0.858	−0.265 *	0.767	—	—	−0.021	0.979
5. Highly Vulnerable and Disengaged	−0.092	0.912	−0.132	0.877	−0.244 *	0.784	0.021	1.021	—	—
**Entity Beliefs**										
1. Moderate Distress with Moderate Functioning	—	—	−0.056	0.945	0.188 *	1.207	−0.055	0.947	−0.404 **	0.667
2. Psychologically Stable but Disengaged	0.056	1.058	—	—	0.245 **	1.277	0.002	1.002	−0.348 *	0.706
3. Flourishing and Engaged	−0.188 *	0.828	−0.245 **	0.783	—	—	−0.243 *	0.784	−0.593 ***	0.553
4. Distressed but Engaged	0.055	1.056	−0.002	0.998	0.243 *	1.275	—	—	−0.350 *	0.705
5. Highly Vulnerable and Disengaged	0.404 **	1.498	0.348 *	1.416	0.593 ***	1.809	0.350 *	1.419	—	—
**Incremental Beliefs**										
1. Moderate Distress with Moderate Functioning	—	—	0.382 **	1.465	−0.435 ***	0.647	−0.324 *	0.723	0.462 *	1.587
2. Psychologically Stable but Disengaged	−0.382 **	0.683	—	—	−0.817 ***	0.442	−0.705 ***	0.494	0.080	1.084
3. Flourishing and Engaged	0.435 ***	1.545	0.817 ***	2.263	—	—	0.111	1.118	0.897 ***	2.452
4. Distressed but Engaged	0.324 *	1.382	0.705 ***	2.025	−0.111	0.895	—	—	0.786 **	2.194
5. Highly Vulnerable and Disengaged	−0.462 *	0.630	−0.080	0.923	−0.897 ***	0.408	−0.786 **	0.456	—	—
**School Connectedness**										
1. Moderate Distress with Moderate Functioning	—	—	0.057	1.059	−1.672 ***	0.188	0.325 *	1.384	1.237 ***	3.444
2. Psychologically Stable but Disengaged	−0.057	0.944	—	—	−1.730 ***	0.177	0.267	1.306	1.179 ***	3.252
3. Flourishing and Engaged	1.672 ***	5.323	1.730 ***	5.638	—	—	1.997 ***	7.365	2.909 ***	18.336
4. Distressed but Engaged	−0.325 *	0.723	−0.267	0.765	−1.997 ***	0.136	—	—	0.912 ***	2.489
5. Highly Vulnerable and Disengaged	−1.237 ***	0.290	−1.179 ***	0.307	−2.909 ***	0.055	−0.912 ***	0.402	—	—

*Note*. *N* = 1729; 119 observations were excluded because of missing auxiliary variables. Each entry compares the row profile with the column reference profile. *Logit B* = unstandardized regression coefficient; *OR* = odds ratio. All 10 covariates were included simultaneously using R3STEP, MLR, and TYPE = MIXTURE COMPLEX (36 classroom clusters). Tests are not adjusted for multiple comparisons. * *p* ≤ 0.05. ** *p* ≤ 0.01, *** *p* ≤ 0.001.
